# Cell-Based Blood Biomarkers for Myalgic Encephalomyelitis/Chronic Fatigue Syndrome

**DOI:** 10.3390/ijms21031142

**Published:** 2020-02-08

**Authors:** Daniel Missailidis, Oana Sanislav, Claire Y. Allan, Sarah J. Annesley, Paul R. Fisher

**Affiliations:** Department of Physiology, Anatomy and Microbiology, La Trobe University, Melbourne, VIC 3086, Australia; dmissailidis@students.latrobe.edu.au (D.M.); O.Sanislav@latrobe.edu.au (O.S.); Claire.Allan@latrobe.edu.au (C.Y.A.); S.Annesley@latrobe.edu.au (S.J.A.)

**Keywords:** myalgic encephalomyelitis, chronic fatigue syndrome, mitochondria, complex V, TORC1, Seahorse respirometry, biomarker, diagnosis, ME/CFS

## Abstract

Myalgic encephalomyelitis/chronic fatigue syndrome (ME/CFS) is a devastating illness whose biomedical basis is now beginning to be elucidated. We reported previously that, after recovery from frozen storage, lymphocytes (peripheral blood mononuclear cells, PBMCs) from ME/CFS patients die faster in culture medium than those from healthy controls. We also found that lymphoblastoid cell lines (lymphoblasts) derived from these PBMCs exhibit multiple abnormalities in mitochondrial respiratory function and signalling activity by the cellular stress-sensing kinase Target Of Rapamycin Complex 1 (TORC1). These differences were correlated with disease severity, as measured by the Richardson and Lidbury weighted standing test. The clarity of the differences between these cells derived from ME/CFS patient blood and those from healthy controls suggested that they may provide useful biomarkers for ME/CFS. Here, we report a preliminary investigation into that possibility using a variety of analytical classification tools, including linear discriminant analysis, logistic regression and receiver operating characteristic (ROC) curve analysis. We found that results from three different tests—lymphocyte death rate, mitochondrial respiratory function and TORC1 activity—could each individually serve as a biomarker with better than 90% sensitivity but only modest specificity vís a vís healthy controls. However, in combination, they provided a cell-based biomarker with sensitivity and specificity approaching 100% in our sample. This level of sensitivity and specificity was almost equalled by a suggested protocol in which the frozen lymphocyte death rate was used as a highly sensitive test to triage positive samples to the more time consuming and expensive tests measuring lymphoblast respiratory function and TORC1 activity. This protocol provides a promising biomarker that could assist in more rapid and accurate diagnosis of ME/CFS.

## 1. Introduction

Myalgic encephalomyelitis/chronic fatigue syndrome (ME/CFS) is amongst the chronic diseases which most adversely affect quality of life [1]. Despite this, diagnosis is often a slow process and relies on internationally varying case criteria, in the absence of a gold standard diagnostic biomarker. The most commonly used definitions require the presence of post-exertional malaise for diagnosis, accompanied by combinations of other, variably presenting symptoms [2,3,4]. Dependence on these cumbersome and varying definitions, which likely capture heterogenous patient populations, has presented a longstanding challenge for patients, clinical practice and the substantial body of ME/CFS research [5]. The identification of robust biomarkers has consequently been one of the most recurring pursuits in the field, since it would allow for more rapid, specific and sensitive diagnoses of patients. While past and ongoing attempts at identifying such a diagnostic solution are numerous, none have yet resulted in a clinically proven biomarker of ME/CFS. It is therefore imperative that new diagnostic tools continue to be sought.

To combat the subjectivity introduced by self-reported symptom scales, objective clinical measures have been explored previously, such as hand grip strength [6] or orthostatic intolerance and standing difficulty [7]. These physical measures have been demonstrated to have value in stratifying ME/CFS patients by disease state/severity and in aiding the separation of patients from healthy subjects. Measures of physical ability may, however, be confounded by other conditions also affecting physical ability. Such tools are, therefore, very useful for the clinical characterisation of patients but are not proven specific to this disease. This highlights the unmet need for specific, molecular biomarkers of ME/CFS.

Accordingly, molecular biomarkers have been pursued across multiple areas of research. For example, the role of the immune system in ME/CFS has been studied for decades, raising cytokines as potential biomarkers of disease [8,9,10,11,12,13,14] whose clinical utility would be aided by their accessibility in blood. Other cytokine studies have utilised cerebrospinal fluid [15,16], the acquisition of which is more invasive than drawing blood, and so could be less amenable to a routine diagnostic test. Despite the number of studies, these reports as a whole are largely inconsistent for individual cytokines [17,18], with initially promising candidates such as transforming growth factor beta 1 being subsequently refuted [19]. Therefore, the utility of cytokine measurements such as an ME/CFS diagnostic tool remains uncertain.

By contrast, studies reporting altered levels of many metabolites in the blood of ME/CFS patients [20,21,22,23,24,25,26,27] are thought to reflect pathway alterations that are largely consistent, albeit with some discrepancies between studies [23]. Such metabolite differences have been proposed as potential candidates for diagnostic blood tests [20,21,22,23,24]. One strength of a diagnostic test which measures multiple molecular parameters is that the likelihood of other diseases displaying the same biochemical pattern is low. However, the techniques utilised in these studies (mass spectrometry or nuclear magnetic resonance spectroscopy) may be subject to rapid fluctuations with patient diet and activity, as well as requiring strict sample acquisition and handling conditions [28,29] and expensive, specialised equipment. These requirements could introduce challenges in a broad clinical setting. Further investigation is therefore warranted, both to address the clinical suitability of blood-based metabolite measurements for diagnosing ME/CFS, and to better understand the remaining inconsistencies. 

The ideal molecular biomarkers would be straightforward to sample and assess, and they also exhibit high sensitivity and specificity. For these reasons, the search for biomarkers has extended to investigating the utility of routine blood pathology tests, but the results are either inconsistent [9,10] or require further study [30]. The recent development of a nanoneedle bioarray to measure the electrical impedance of ME/CFS peripheral blood mononuclear cells (PBMCs) in plasma is highly promising, but requires identification of the underlying mechanism and proven specificity for ME/CFS [31]. Therefore, the need for a simple blood-based biomarker remains currently unfulfilled.

It has long been suspected that mitochondrial dysfunction might play a role in the cytopathology, but the small number of direct investigations of this had produced confusing, contradictory results. We recently reported both specific mitochondrial dysfunction and cellular signalling dysregulation in ME/CFS patient blood-derived lymphoblasts, as well as an associated reduction in the viability in culture of ex vivo, stored PBMCs from patients versus healthy controls [32]. We now examine the discriminatory utility of these differences as components of a potential, blood-based diagnostic test.

## 2. Results

### 2.1. ME/CFS and Patient Blood Samples Can be Distinguished by the Viability in Culture of Frozen Peripheral Blood Lymphocytes

We reported, in an accompanying paper, that after recovery from frozen storage, PBMCs from ME/CFS patients die markedly faster in culture than those from healthy controls [32]. A time course revealed that the percentage of dead cells, and the difference between patients and controls, increased dramatically from 24 to 72 h of culture. To determine the utility of this difference in viability for distinguishing patient and control PBMCs, we used both linear discriminant analysis and logistic regression (Table 1). Both types of analysis performed identically using lymphocyte death rates at 48 h in culture, with an overall error rate close to 20% (Table 1). However, in both cases the relative frequencies of false positives and false negatives were skewed in favour of a low fraction ( < 10%) of ME/CFS patients being incorrectly classed as “controls”, while about 40% of the controls were incorrectly classed as ME/CFS.

We also used the percentage of dead PBMCs in culture at all three time points (24, 48 and 72 h) in multiple logistic regression or linear discriminant analysis to determine if that approach would produce better discrimination between patients and controls (Appendix A Table A1). The overall error rate was again close to 20%, although the frequency of false negatives was slightly higher and the frequency of false positives was slightly lower than when using the 48 h death rate alone. The results from the linear discriminant and logistic regression analyses were again almost identical and showed that the percentage of dead PBMCs after 48 h culture performed just as well as regressing the viability against incubation time. The single time point assay would be simpler and cheaper to use for clinical purposes. We conclude that PBMC isolation, frozen storage and subsequent testing for viability after 48 h in culture provides a reliable biomarker for distinguishing ME/CFS and healthy control blood samples.

**Figure 1:**
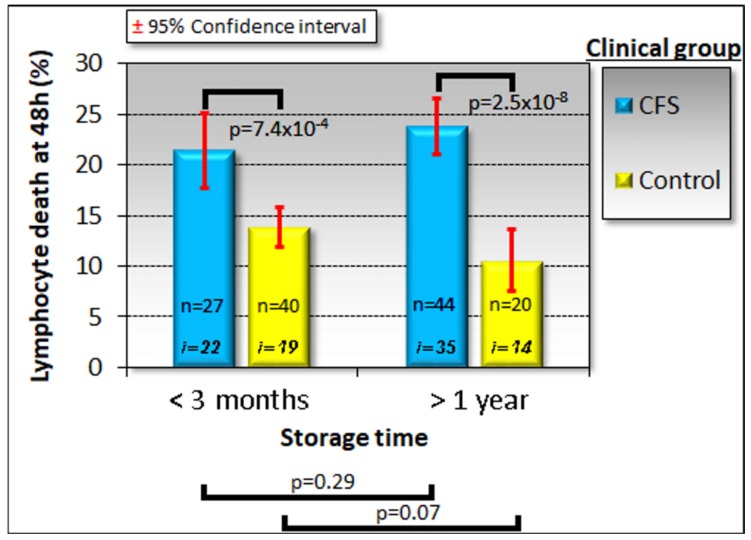
Time in frozen storage has no effect on the viability of lymphocytes after recovery and incubation in culture medium for 48 h. Some individuals were sampled on more than one occasion and some samples were tested at more than one storage time point using separately frozen aliquots. The sample sizes indicated (n) are the number of frozen aliquots that were tested from the number of individuals shown (*i*). Since the storage time had no effect in either ME/CFS patient or control samples, multiple samples tested from the same individual were averaged for subsequent analysis. The fraction of dead cells was greater in ME/CFS lymphocytes. Significance probabilities shown are from pairwise *t* tests of the difference in means.

During the course of our study, before being used for lymphoblast isolation or biochemical studies, PBMCs were kept frozen at −80 °C for differing lengths of time ranging from a few days to almost 3.5 years. It has been previously reported that PBMCs remain viable for long periods in frozen storage under similar conditions [33]. Because biomarker stability is important in the face of varying circumstances, such as the time of frozen storage of the sample, we verified that the death rate of PBMCs recovered from frozen storage and kept in culture for 48 h was not significantly altered by the time spent in storage (Figure 1).

**Figure 2:**
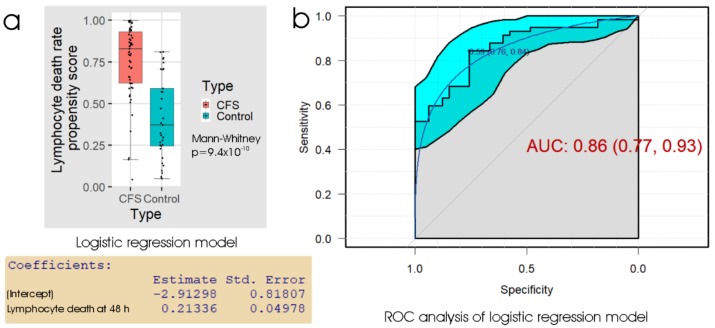
Logistic regression and ROC analysis of the percentage of dead lymphocytes after 48 h post-storage culture. (**a**) Box plot showing the distribution of the propensity score in logistic regression of the sample type against the fraction of dead lymphocytes observed after recovery from frozen storage and 48 h culture. The resulting regression coefficients are as indicated. The boxes show the median and the 25^th^ and 75^th^ percentiles, so that the height of the box is the interquartile range (IQR). The whiskers extend to the most extreme observations (largest and smallest) falling within ± 1.5 × IQR of the box. The ME/CFS and control sample sizes were 57 and 33 individuals, respectively. The Mann–Whitney significance probability tests the hypothesis that the scores in ME/CFS samples are greater than in control samples. Scores greater than 0.5 lead to classification of a sample as ME/CFS in the “confusion matrix”. Other relevant statistics showing that the logistic regression model provided a good fit to the data were: Hesmer–Lemeshow goodness of fit = 0.11; Pseudo R^2^ = 0.59; χ^2^ *p* = 1.9 × 10^−7^. (**b**) ROC analysis of the propensity score, plotting sensitivity (proportion of true positives) against specificity (proportion of true negatives) with 95% confidence limits (cyan shading). The fractional area under the curve (AUC) is shown with 95% confidence limits. The “best” threshold for the propensity score (0.59) is shown, together with the specificity (0.76) and sensitivity (0.84) at that threshold (in parentheses).

To further assess the biomarker potential of measuring the death rate of frozen lymphocytes after recovery and culture for 48 h, we conducted ROC analysis of the propensity score from the logistic regression (Figure 2). The results showed that using the “best” threshold (maximising the sum of the sensitivity and specificity) of 0.59 for the propensity score is effective, and this corresponded to a threshold of 16% in the 48 h lymphocyte death rate. The specificity at this threshold was 76% (24% false positives) and the sensitivity was 84% (16% false negatives). As anticipated, this represents a similar overall performance, but a smaller difference between sensitivity and specificity, compared to the thresholds used by either linear discriminant analysis or logistic regression in Table 1. The area under the ROC curve (AUC), a measure of reliability, indicated that the 48 h lymphocyte death rate could be a useful clinical test, bearing in mind that the result can be obtained from a small blood sample within a few days. For comparison with another chronic disease, clinical diagnosis of idiopathic Parkinson’s disease (PD) by a neurologist is able to achieve a reliability of about 70% with high sensitivity (ca. 90%), but low specificity (ca. 60%) (relative to postmortem neuropathological diagnosis), the low specificity being partly due to confusion with similar diseases [34,35]. More reliable diagnosis of PD can be achieved by movement disorder specialists.

### 2.2. Immortalised Lymphocytes from ME/CFS and Patient Blood Samples Can be Distinguished by Mitochondrial and Cellular Respiratory Dysfunction 

**Figure 3:**
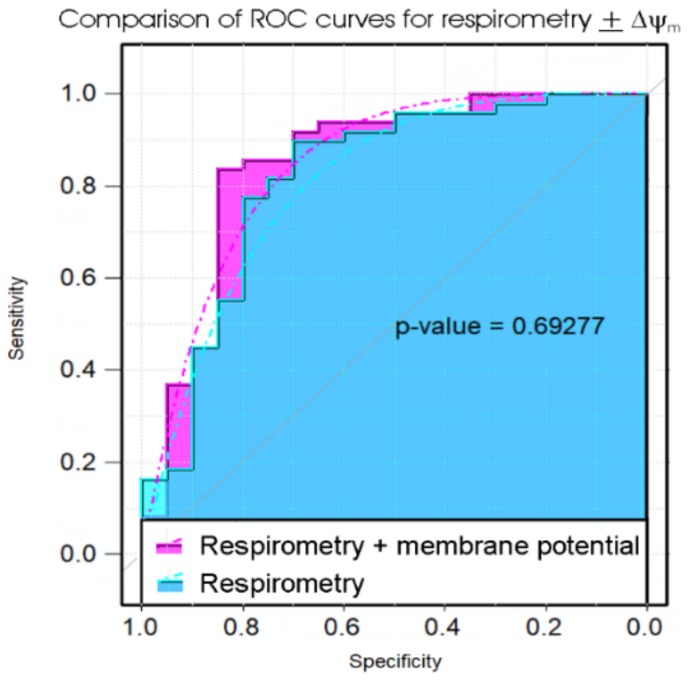
Comparison of ROC curves of propensity scores in logistic regression of five key parameters of lymphoblast respiration with or without measures of the mitochondrial membrane potential. The significance probability of a bootstrap test of the difference between the ROC curves (Robin et al., 2011) is indicated [36]. The addition of the extra laboratory assay (for mitochondrial membrane potential, ΔΨ_m_) did not significantly improve the diagnostic value of the test.

Although the PBMC death rate in culture provides a simple and potentially useful test, the diagnosis of ME/CFS would benefit from even higher sensitivity and specificity than this assay was able to provide. We previously showed that immortalised lymphocytes (lymphoblasts) from ME/CFS patients exhibit significant abnormalities in mitochondrial and cellular respiratory function. The key measures of mitochondrial and cellular respiratory function that were changed in patients, compared to controls, were the mitochondrial membrane potential (ΔΨ_m_), the rate of O_2_ consumption (OCR) by ATP synthesis and the proton leak (as fractions of the basal respiration rate), the maximum OCR by uncoupled mitochondria, the uncoupled activity of Complex I and the non-mitochondrial OCR (a surrogate measure of overall metabolic rate). We used these parameters in discriminant analysis and multiple logistic regression to determine their efficacy in distinguishing ME/CFS and control patients. Of these measures, all but ΔΨ_m_ are obtained from the same respirometry experiments. For this reason, it was worthwhile determining if ΔΨ_m_ provided significant additional discriminatory power in the tests. We therefore compared the results using the five respiration measures with and without ΔΨ_m_. The results (Table 2, Figure 3) showed there was only a small extra benefit in using the assay of ΔΨ_m_ in addition to the respirometry—the “confusion” matrices revealed slightly higher test specificity when the ΔΨ_m_ was included, but the sensitivities were identical and ROC curves were not significantly different.

**Figure 4:**
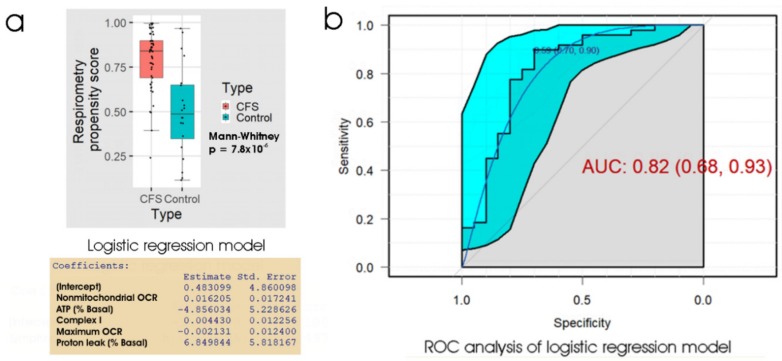
Logistic regression and ROC analysis of lymphoblast respiration as an ME/CFS biomarker. (**a**) Box plot showing the distribution of the propensity score from logistic regression of participant type against 5 key respiratory parameters. The resulting regression coefficients are as indicated. The boxes show the median and the 25^th^ and 75^th^ percentiles, so that the height of the box is the interquartile range (IQR). The whiskers extend to the most extreme observations (largest and smallest) falling within ± 1.5 × IQR of the box. The ME/CFS and control sample sizes were 49 and 20 individuals, respectively. The Mann–Whitney significance probability tests the hypothesis that the scores in ME/CFS samples are greater than in control samples. Each point represents a single individual. Scores greater than 0.5 lead to classification of a sample as ME/CFS in the “confusion matrix”. Other relevant statistics showing that the logistic regression model provided a good fit to the data were: Hesmer–Lemeshow goodness of fit = 0.14; Pseudo R^2^ = 0.56; χ^2^ *p* = 6.7 × 10^−4^. (**b**) ROC analysis of the propensity score, plotting sensitivity (proportion of true positives) against specificity (proportion of true negatives) with 95% confidence limits (cyan shading). The fractional area under the curve (AUC) is shown with 95% confidence limits. The “best” threshold for the propensity score (0.59) is shown, together with the specificity (0.70) and sensitivity (0.90) at that threshold (in parentheses).

Once again, the linear discriminant analysis and logistic regression methods produced almost identical results. Figure 4 shows the results of ROC analysis and a box plot of the propensity scores from logistic regression of the five key respiratory parameters. At the “best” threshold of the regression propensity score, the false positive error rate was 30% and the false negative error rate was 10%. With an AUC of 0.82, the ROC curve (for the ability of the lymphoblast respirometry measures to discriminate between ME/CFS patients and controls) was not significantly different from that (AUC = 0.86) obtained using the 48 h lymphocyte death rate (*p* = 0.60). We conclude that for diagnostic purposes, the performance of the two tests (48 h lymphocyte death rate and lymphoblast respirometry) is very similar and either could be used as a biomarker for ME/CFS.

### 2.3. Immortalised Lymphocytes from ME/CFS and Patient Blood Samples Can be Distinguished by the Phosphorylation State of 4E-BP1, a TORC1 Kinase Substrate

The foregoing results showed that good biomarkers for ME/CFS are provided by both the death rates of stored lymphocytes after 48 h in culture medium and the respiratory function of cultured lymphoblasts derived from them. In both cases, the optimum thresholds were found to discriminate ME/CFS from control cells with a reliability better than 80% (AUC), with an overall error rate of less than 20%. However, in both cases, the errors at these “optimal” thresholds were not proportionately distributed between the patients and controls. Thus, although more than 90% of patient samples were correctly identified as such (high sensitivity), the specificity was relatively low in that more than 40% (logistic regression) of the control samples were also classed incorrectly as being ME/CFS. Using ROC analysis to find the best threshold to minimise the error rates resulted in only small improvements. We, therefore, conducted similar analysis to determine whether the elevated TORC1 activity in lymphoblasts might perform better as a biomarker of ME/CFS than the lymphocyte death rates or the lymphoblast respiratory dysfunction. 

**Figure 5:**
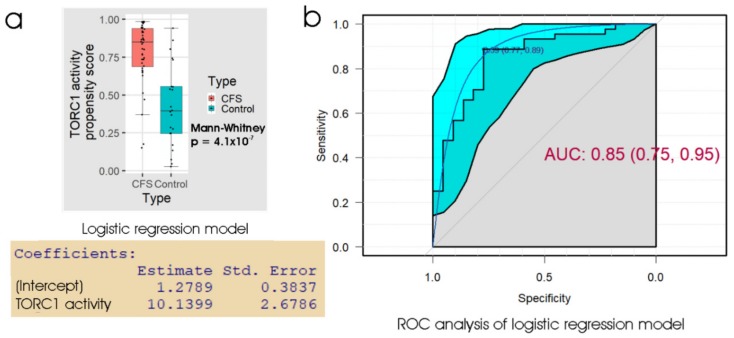
Logistic regression and ROC analysis of lymphoblast TORC1 activity. (**a**) Box plot showing the distribution of the propensity score from logistic regression of participant type against the logarithm of the relative TORC1 activity, measured as phosphorylation state of 4E-BP1 (a specific cellular target of TORC1). The resulting regression coefficients are as indicated. The boxes show the median and the 25^th^ and 75^th^ percentiles, so that the height of the box is the interquartile range (IQR). The whiskers extend to the most extreme observations (largest and smallest) falling within ± 1.5 × IQR of the box. The ME/CFS and control sample sizes were 44 and 22 individuals, respectively. The Mann–Whitney significance probability tests the hypothesis that the scores in ME/CFS samples are greater than in control samples. Each point represents a single individual. Scores greater than 0.5 lead to classification of a sample as ME/CFS in the “confusion matrix”. Other relevant statistics showing that the logistic regression model provided a good fit to the data were: Hesmer–Lemeshow goodness of fit = 0.53; Pseudo R^2^ = 0.60; χ^2^ *p* = 1.9 × 10^−7^. (**b**) ROC analysis of the propensity score, plotting sensitivity (proportion of true positives) against specificity (proportion of true negatives) with 95% confidence limits (cyan shading). The fractional area under the curve (AUC) is shown with 95% confidence limits. The “best” threshold for the propensity score (0.59) is shown, together with the specificity (0.77) and sensitivity (0.89) at that threshold (in parentheses).

The results (Table 3) showed that the lymphoblast TORC1 activity assay also produced an overall error rate of about 20%, similar to the other assays. In ROC analysis (Figure 5) the AUC was 0.85 and at the “best” threshold, the specificity was 0.77 and sensitivity was 0.89. This suggests, and ROC curve comparisons confirmed, that the TORC1 activity assay was no better a discriminator than either the 48 h lymphocyte death rate (*p* = 0.26) or the lymphoblast respiratory dysfunction (*p* = 0.68).

### 2.4. Combining Measures of Frozen Lymphocyte Death Rate, Lymphoblast Mitochondrial Dysfunction and Lymphoblast TORC1 Signalling Discriminates ME/CFS and Patient Blood Samples with High Accuracy

**Figure 6:**
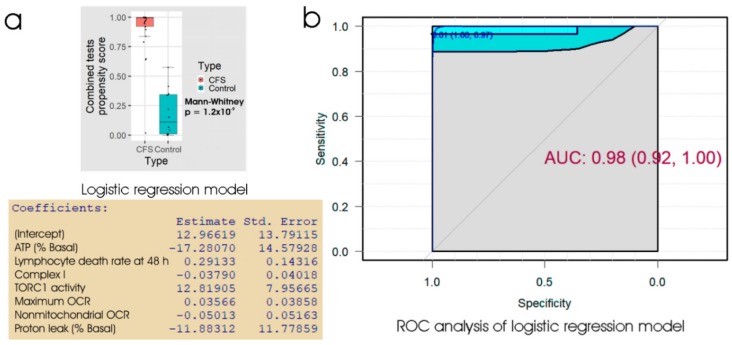
Logistic regression and ROC analysis of combined tests for lymphocyte death in culture, lymphoblast respiration and lymphoblast TORC1 activity. (**a**) Box plot showing the distribution of the propensity score from logistic regression of the percentage of dead lymphocytes after 48 h in culture, 5 key parameters of lymphoblast respiration and the TORC1 activity. The resulting regression coefficients were as indicated. The boxes show the median and the 25^th^ and 75^th^ percentiles, so that the height of the box is the interquartile range (IQR). The whiskers extend to the most extreme observations (largest and smallest) falling within ± 1.5 × IQR of the box. The Mann–Whitney significance probability tests the hypothesis, that the scores in ME/CFS samples are greater than in control samples. Each point represents a single individual. Scores greater than 0.5 lead to classification of a sample as ME/CFS in the “confusion matrix”. Other relevant statistics showing that the logistic regression model provided a good fit to the data were: Hesmer–Lemeshow goodness of fit = 0.77; Pseudo R^2^ = 0.87; χ^2^ *p* = 2.9 × 10^−6^. (**b**) ROC analysis of the propensity score, plotting sensitivity (proportion of true positives) against specificity (proportion of true negatives) with 95% confidence limits (cyan shading). The fractional area under the curve (AUC) is shown with 95% confidence limits. The “best” threshold for the propensity score (0.61) is shown, together with the specificity (1.0) and sensitivity (0.97) at that threshold (in parentheses).

All three assays tested in the preceding sections (the lymphocyte death rate, the lymphoblast respirometry or lymphoblast TORC1 activity) provided good biomarkers for ME/CFS, but none was significantly better than the others, and none were perfect discriminators between patients and controls. In pursuit of an even better discriminator, we combined all three of these assays to produce a single measure by either linear discriminant analysis or logistic regression. The results (Table 4, Figure 6) showed that the combination of all three assays was able to discriminate between ME/CFS patients and healthy controls with about 95% accuracy in our sample. Whereas the three individual tests described in the preceding sections produced ROC curves with broad confidence limits, the 95% confidence limits for the ROC curve using the combined tests were much narrower i.e., the estimated errors were much smaller. At the “best” threshold of 0.61 in the ROC analysis, the specificity was 100% and the sensitivity was 97%. The combination of lymphocyte 48 h death rate, lymphoblast respiratory dysfunction and lymphoblast TORC1 activity may thus provide a reliable discriminator between ME/CFS and control samples.

### 2.5. A protocol that Combines a Screening Test Using Lymphocyte Death Rates and Confirmatory Tests of Respiratory Function and TORC1 Activity

The results in the preceding sections showed that three different cell-based blood tests (lymphocyte death rate, lymphoblast respiratory function and lymphoblast TORC1 activity) can individually provide biomarkers of ME/CFS compared to healthy controls. Of these, the lymphocyte death rate in culture could potentially provide a relatively quick test in the clinical setting, since it requires only separation and storage of the PBMCs from a blood sample followed by incubation in culture medium for two days. It exhibited high sensitivity but low specificity for ME/CFS and thus, would be well suited for screening purposes. However, when combined with the lymphoblast respiratory function and TORC1 activity tests, it provided about 95% sensitivity and specificity, a feat not achieved by any of the three assays alone. These outcomes suggest a testing protocol in which an initial assay of lymphocyte death rate would be followed, in the case of positive results, by lymphoblast isolation, Seahorse respirometry and TORC1 activity assay. Because it involves seven different biochemical, mitochondrial and cellular parameters, such a combination of tests may provide greater specificity vís a vís other conditions (e.g., thyroxin deficiency) that may cause chronic fatigue. However, that remains to be tested in future work.

**Figure 7:**
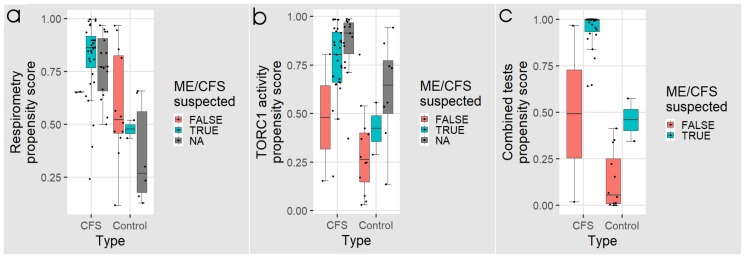
Box plots showing the distribution of logistic regression propensity scores for individuals from whom the fraction of dead lymphocytes after 48 h in culture would have tested as “ME/CFS suspected” or “ME/CFS not suspected”. NA refers to individuals for whom we have respirometry or TORC1 activity but not lymphocyte death rates. Propensity scores from (**a**) five key parameters of lymphoblast respiration, and (**b**) the TORC1 activity are plotted for each individual. In panel (**c**) the propensity scores from all three tests combined are shown. The regression coefficients were as indicated in Figure 4, Figure 5 and Figure 6, respectively. The boxes show the median and the 25^th^ and 75^th^ percentiles, so that the height of the box is the interquartile range (IQR). The whiskers extend to the most extreme observations (largest and smallest) falling within ± 1.5 × IQR of the box. Each point represents a single individual. Scores greater than 0.5 lead to classification of a sample as ME/CFS in the corresponding “confusion matrix”.

To determine the potential efficacy of this protocol, we simulated it by subdividing our samples into subgroups, based on whether ME/CFS would be suspected or not from the lymphocyte death rates, using the “best” threshold from the ROC analysis as the criterion. Figure 7 and Table 5 and Table 6 show the results for these individual subgroups in the three assays. Of the 29 ME/CFS patients for whom we have data for all three assays, 27 would have been classed as “ME/CFS suspected” based on lymphocyte death rates, and all of these would have been subsequently confirmed by the combined tests (Figure 7c, Table 5). Of the remaining two patients classed as “ME/CFS not suspected”, one was “ME/CFS positive” in the combined tests. This individual would have been misclassified, if the combined tests had not been done and thus would have been missed in a protocol using the lymphocyte death rate alone as a screen. The remaining individual was misclassified as not having ME/CFS, even using the combined tests. 

Amongst the smaller number of control participants, 12 would have been correctly classed as “ME/CFS not suspected” based on screening using the lymphocyte death rate in culture. Of the remaining two individuals suspected as having ME/CFS, the combined tests would have classed one as “healthy” and one only would have been misclassified as ME/CFS (Figure 7c, Table 5).

Because of limitations on the supply of lymphocytes, 23 individuals were not tested for lymphocyte death rates. However, they were tested for lymphoblast TORC1 activity and all ME/CFS patients but one were also tested in the lymphoblast respirometry assay. As was the case with the full data set, both of these assays exhibited high sensitivity for ME/CFS, but poor specificity in this subgroup (Table 6, Figure 7a,b).

## 3. Discussion

The diagnosis of ME/CFS currently requires patients to exhibit, for at least six months, the hallmark symptoms of chronic fatigue and post-exertional malaise that cannot be explained by other conditions. For patients, physicians and the health care system, this diagnosis—that depends on exclusion of other illnesses—is a long, frustrating and potentially expensive process. Suitable diagnostic biomarkers have not yet been identified, although a recent report of an altered electrical impedance response to salt stress in patient lymphocytes looks highly promising [31]. We reported, in an accompanying paper, that ex vivo lymphocytes from ME/CFS patients exhibit an elevated death rate in culture after recovery from frozen storage and that lymphoblastoid cell lines (lymphoblasts) isolated from them exhibit multiple mitochondrial and cellular stress signalling abnormalities. These abnormalities were measured in four different laboratory tests—lymphocyte death rates, lymphoblast mitochondrial membrane potential, lymphoblast mitochondrial respiratory function and lymphoblast TORC1 signalling activity. The mitochondrial respiratory dysfunction included changes in several key measures of mitochondrial activity—reduced mitochondrial membrane potential and lower efficiency of ATP synthesis, increased “proton leak” relative to basal metabolic rate, and elevated maximum rates of respiration and Complex I activity. Our purpose here was to determine if these abnormalities in cells derived from ME/CFS patient blood samples could be used as biomarkers of disease.

The results from the linear discrimination and logistic regression analyses were almost identical for all of the biomarkers we used here. This is not surprising as they are related methods and have produced similar outcomes in other studies [37]. However, logistic regression is considered to be more robust against departures of the input data from normality, equality of variances and the presence of outliers. To confirm that the proposed biomarkers would be effective in correctly diagnosing ME/CFS in new samples that were not part of the original analysis, we randomly selected 80% of our sample for use as a training set, while the remaining 20% was used as a test data set. Despite the fact that the smaller training set makes the parameter estimates and threshold determination less accurate, very similar results to those obtained with the full data set were obtained in all of our assays and all performed as well with the test data set as with the training set (Appendix A Table A2). We also trialed and found that support vector machine and neural network models produced very similar outcomes for all of the biomarkers we tested here. These results give confidence that the proposed biomarkers could be usefully deployed for diagnostic purposes.

Of the biomarker tests we examined, the lymphocyte death rates are the simplest and cheapest, and would provide the quickest result in a clinical setting. We found that the fraction of dead lymphocytes after frozen storage and culture for 48 h could distinguish between ME/CFS and control samples with a high sensitivity but only modest specificity. Although ex vivo lymphocyte death rates were elevated compared to controls on all three days of culture after recovery from frozen storage, assessing the proportion of dead cells in the culture at more than one time point did not improve the discriminatory value of the test. Of the remaining three types of test, the measurement of mitochondrial membrane potential produced no significant improvement over the respirometric assay of mitochondrial function, so we discarded its use in the remaining analysis. Both the respiratory function assay and the TORC1 activity assay produced similar results to the lymphocyte death assay—high sensitivity combined with low specificity. However, when all three tests were combined using multiple logistic regression, we found very high sensitivity and specificity, with only two from a total of 43 samples (one patient and one control) being misclassified. Except for the recently reported lymphocyte impedance response to salt stress [31], this combination of three tests (lymphocyte death rate, lymphoblast mitochondrial respiratory function and lymphoblast TORC1 activity) thus achieves better accuracy and reliability (sensitivity, specificity and AUC) for discriminating ME/CFS than has been previously reported using other blood-based molecular tests [20,21,22,23].

Because of the time, expertise and expense associated with lymphoblast isolation and testing, we explored whether a two-stage test would be suitable. Our results suggested a test protocol which can discriminate between ME/CFS patients and healthy individuals with near-perfect accuracy. In this protocol, the frozen lymphocyte viability after 48 h in culture would be used as an initial screening test. With its high sensitivity, low cost and speed, it requires only a small blood volume allowing most of the sample to be utilised for subsequent confirmatory tests. We have also demonstrated that the test result is stable over long periods of frozen storage. These practical advantages lend great value to the protocol in a broader clinical context and set it apart from methods which would proceed directly to highly specialised testing. Applied to the subset of 43 samples for which we have results from all three tests, this initial screening would have failed to detect only one patient who would have tested positive if all three tests had been combined. One patient and one control sample were misclassified, regardless of using the staged protocol or the combined tests, so that the staged protocol would have misclassified only one additional patient. We therefore propose that a staged protocol combining the clinical suitability of the lymphocyte screening step and the discriminatory power of the entire protocol provides a promising diagnostic biomarker for ME/CFS. The patient and their clinician could choose on the basis of the results from the lymphocyte death rate test, whether or not to proceed with the slower, more expensive confirmatory tests.

One limitation of the viability assay we used in this work, Trypan Blue staining, is that although it is very fast and simple in principle, it requires microscopy and cell counting by a person skilled in the art. It would be valuable in future work to determine if some of the many other cell viability assays that are commercially available might lend themselves more readily to use in a reproducible way by less skilled personnel. This would facilitate higher throughput and greater accuracy in the results in a clinical setting.

A further limitation of the testing protocol suggested here is that it has so far been fully tested on a relatively small sample of 29 patients and 14 controls for whom we have a complete data set from all tests. The testing protocol here will need to be refined using much larger samples in order to both validate it and to determine more accurately the weighting coefficients that should be applied in the logit function (from the logistic regression). At this stage, we can nonetheless conclude that the cell-based blood biomarkers used here are amongst the most promising candidates so far identified for potential use in diagnosing ME/CFS.

A limitation of all biomarkers so far proposed for ME/CFS is that it is not yet known how specific they are vís a vís other illnesses which cause chronic fatigue and/or post exertional malaise and with which ME/CFS may potentially be confused. It would be useful to examine the viability levels of frozen lymphocytes in similar diseases to assess how specific the elevated death rate of frozen lymphocytes is to ME/CFS patients. It has been documented previously, that frozen PBMC viability is also reduced in paediatric Dengue fever [38]. However, the strength of the frozen lymphocyte viability test is its high sensitivity as a screening step to successfully detect ME/CFS individuals and correctly triage true-positive ME/CFS samples towards the subsequent confirmatory tests. It seems possible that the number and specific biological nature of these subsequent measurements means that the resulting molecular read-out is likely to be unique to ME/CFS, particularly in combination with the patient’s clinical history. To be confused with ME/CFS in the confirmatory tests of mitochondrial respiratory function and TORC1 activity, other illnesses would not only need to cause reduced viability of frozen lymphocytes, they would also need to confer the same pattern of molecular abnormalities upon the derived lymphoblasts—decreased Complex V efficiency, elevated proton leak as a proportion of basal metabolic rate, as well as increases in maximum respiratory capacity, Complex I activity, nonmitochondrial oxygen consumption and TORC1 activity. It is worth noting that lymphoblasts from patients with Parkinson’s disease, another complex chronic disorder, exhibit a quite different pattern of abnormalities related to mitochondrial function [39]. Of course, Parkinson’s disease patients are unlikely to be confused clinically with ME/CFS patients in the first place. Thus, although the combination of cellular and molecular phenotypes reported here may well be unique to ME/CFS, it will be essential in future work to determine its specificity in relation to other, similar diseases. 

Despite the clarity of the differences we have observed in frozen lymphocyte viability, the underlying reason for the elevated death of ME/CFS lymphocytes after storage remains undetermined. Impaired mitochondrial respiratory function, including Complex V impairment, has long been known to result in apoptotic cell death in ex vivo lymphoid cells [40]. However, we have not demonstrated whether the lymphocyte death we observe is apoptotic or whether it is one of the other known forms of eukaryotic cell death. Whatever the specific cell death pathway involved, it may reflect an inability of ME/CFS patient cells to adequately respond to cellular damage or stress. In this case, such an insult could be introduced by freezing, which is well understood to damage biological systems by the formation of ice crystals, but has also been documented to specifically affect PBMC viability, function, and expression of stress response genes [41,42]. While the elevated death rate of the ME/CFS lymphocytes could reflect a greater mechanical susceptibility to immediate structural damage by freezing, there remains another possibility. Compared with controls, the number of dead ME/CFS lymphocytes continued to increase at a faster rate than the controls over multiple days in culture. This suggests that underlying and ongoing cytopathological processes could be contributing towards cell death in culture of previously frozen lymphocytes.

## 4. Materials and Methods

### 4.1. Participant Cohort

Participants were assessed and samples collected by trained staff from La Trobe University and the CFS Discovery Clinic, Melbourne, Australia. Testing was carried out at participant homes when the severity of illness precluded patients from travelling. Participants were selected using the Canadian Consensus Criteria [4] assessed for postorthostatic tachycardia syndrome comorbidity, and asked to complete the Depression, Anxiety and Stress Scale questionnaire and the Epworth Sleepiness Scale questionnaire. ME/CFS-specific severity assessments were also conducted using Richardson and Lidbury’s weighted standing time [7]. For PBMC isolation, 15 mL of blood was taken per participant in heparin-treated vacutainer tubes (BD). Patients with other known reasons for fatigue were excluded. 

For most of the work reported here, we used the same age- and gender-matched participant groups as reported in the accompanying paper [32]. This cohort included 51 ME/CFS patients (86% female, median age 51, age range 26–71) and 22 healthy controls (68% female, median age 43, age range 21–67). However, because of limitations on the supply of lymphocytes, we were able to assay lymphocyte death rates in culture only in a subset of these participants—35 patients (89% female, median age 52, age range 26–71) and 14 controls (71% female, median age 42, age range 21–58). Furthermore, these lymphocyte samples had been stored for significant time periods ( > one year). Therefore, we took the opportunity afforded by a recent influx of new participant samples to include more recently obtained samples prepared in the three month period prior to submission of this manuscript. This additional cohort contained 13 new ME/CFS participants (85% female, median age 38, age range 22–70) and 19 new control individuals (42% female, median age 29, age range 19–55). While the age distribution in the new cohort was not significantly different between the patient and control groups (Fisher exact test, *p* > 0.1), the gender proportions were significantly different (*p* = 0.02). As a consequence, the gender proportions in the total cohort also differed significantly (*p* = 0.002) between the patient and control groups used for the lymphocyte death rates. Nevertheless, this additional cohort allowed us to verify our previous finding [32], that the lymphocyte death rates in culture were not age- or gender-dependent, and to confirm published reports that lymphocyte viability is stable for long periods in frozen storage [33,43].

Participants were recruited and samples obtained with approvals by the Australian National University Human Research Ethics Committee (Reference 2015/193, accepted by the La Trobe University Human Ethics Committee on 26 February 2016) and the La Trobe University Human Ethics Committee (Reference HEC19316, approved on 26 August 2019).

### 4.2. PBMC Isolation from Blood Sample

This method has been described in an accompanying paper [32]. Briefly, lymphocytes were isolated by Ficoll-Paque density centrifugation and counted. For immortalization, 5 × 10^6^ cells were set aside and resuspended in 5 mL Roswell Park Memorial Institute (RPMI) 1640 without L-glutamine (Life Technologies) supplemented with 1X Glutamax (Life Technologies, Carlsbad, California, United States), 10% fetal bovine serum (FBS) and 1% Penicillin/Streptomycin. Excess lymphocytes were separated into aliquots of 5 × 10^6^ cells, harvested and resuspended in 250 µL of Recovery™ Cell Culture Freezing Medium (Life Technologies, Carlsbad, California, United States) and stored at 0 °C.

### 4.3. Immortalisation of Lymphocytes

One mL of culture supernatant from B95.8 cells expressing Epstein-Barr virus (EBV) was added to 5 × 10^6^ cells in 5 mL RPMI 1640. Per well, 150 µL of the mix was seeded in a 96-well U-bottom plate, then incubated for one hour within a humidified 5% CO_2_ incubator at 37 °C. A final concentration of 500 ng/mL Cyclosporin A (Sigma, St. Louis, MO, USA) was then added to each well. Cultures were fed weekly by replacing half of the medium with the same formulation, without disturbing the cells. This process was repeated over a period of approximately three weeks until the cells were confluent and growing rapidly, after which the lymphoblast cultures were processed as described in the following section.

### 4.4. Lymphoblast Cultures

Confluent lymphoblasts were transferred to T25 flasks in growth medium (Minimum Essential Medium α (Life Technologies, Carlsbad, California, United States), supplemented with 10% FBS and 1% Penicillin/Streptomycin), where they were cultured within a humidified 5% CO_2_ incubator at 37 °C. Lymphoblast storage in Recovery™ Cell Culture Freezing Medium at −80 °C has been previously described in detail [32].

Prior to commencing experiments, lymphoblast lines were cultured over as short a time and as few passages (2–5) as possible. For a set of triplicate, independent experiments, harvest, assay and conduct of experiments occurred over approximately one week. Two immortalised lymphoblast cell lines created from healthy donor blood were utilised as internal controls to normalise for variation between experiments where appropriate.

### 4.5. Viable Cell Counts

Lymphoblast or lymphocyte (PBMC) viable counts for all applications were determined by staining with Trypan blue (Thermo-Fisher Scientific, Waltham, Massachusetts, United States) prior to haemocytometer cell counting. Trypan blue-stained cells were counted as dead and unstained, intact cells as viable. For the unimmortalised lymphocyte viability measurements over time, frozen aliquots were thawed in a 37 °C water bath, pelleted at 1000 × *g* for 2 min and resuspended in 1 mL RPMI 1640 without L-glutamine supplemented with 1X Glutamax, 10% FBS and 1% Penicillin/Streptomycin. The cells were then washed at 1000 × *g* for 2 min and resuspended in fresh medium of the same formulation. They were then seeded in 96-well U-bottom plate at a density of 1 × 10^6^ cells/mL and kept in a humidified 5% CO_2_ incubator at 37 °C over the course of the experiment. Each well was mixed gently by pipette before sampling to ensure counting of a homogeneous cell suspension.

### 4.6. Mitochondrial Stress Test (Seahorse Respirometry)

Oxygen consumption rates (OCR) of 8 × 10^5^ viable PBMCs or lymphoblasts per well were measured using the Seahorse XFe24 Extracellular Flux Analyser with Seahorse XF24 FluxPaks (Agilent Technologies, Chicopee, Massachusetts, USA). Immortalised lymphoblasts were cultured in 3 mL growth medium per well in 6-well Costar plates prior to Seahorse experiments. Seahorse assays were carried out as previously described in detail [39]. Oxygen consumption rates (OCR in pmol/min) were measured (basal OCR) prior to and after successive injection of 1 µM oligomycin (ATP synthase inhibitor), 1 µM CCCP (carbonyl cyanide m-chlorophenyl hydrazone, an uncoupling protonophore), 1 µM rotenone (Complex I inhibitor) and 5 µM antimycin A (Complex III inhibitor). From the resulting data, we determined the OCR associated with respiratory ATP synthesis (oligomycin-sensitive), the maximum OCR in CCCP-uncoupled mitochondria and the rotenone-sensitive OCR attributable to uncoupled Complex I activity, the antimycin-sensitive Complex II/III activity, the OCR by mitochondrial functions (e.g., protein import) other than ATP synthesis that are Δψm-driven (so-called ‘proton leak’), non-respiratory oxygen consumption (e.g., by cellular and mitochondrial oxygenases and oxidases), and the respiratory ‘spare-capacity’ (excess capacity of the respiratory electron transport chain that is not being used in basal respiration).

### 4.7. 4E-BP1 Phosphorylation Levels (TORC1 Activity)

TORC1 activity in ME/CFS lymphoblast lysates was measured as previously described [32] using a time-resolved FRET-based multiwell plate assay of the phosphorylation state of 4E-BP1, a major TORC1 substrate (Cisbio Bioassays, Codolet, France).

Lymphoblasts were harvested, resuspended in growth medium at 2.75 × 10^5^ cells/mL and plated in four replicates at 5 × 10^4^ cells/well in a 96-well plate. Cells were incubated at 5% CO_2_ / 37 °C for 2 h, with two of the replicates subjected to TOR inhibition by 0.5 µM TORIN2. Lysis buffer was added to each well as per manufacturer instructions and the plate mixed on an orbital shaker for 40 min before plating each sample into a 384 well white plate (Corning, New York, USA)—incorporating various controls and antibody mix (anti- 4E-BP1 antibody labelled with d2 acceptor, and anti-phospho-4E-BP1 antibody labelled with Eu^3+^-cryptate donor) according to manufacturer instructions. After a 2 h incubation at room temperature the plate was scanned by the Clariostar plate reader (BMG, Ortenberg, Germany) and the ratio of the FRET signal from anti-phospho-4E-BP1 antibody to the donor fluorescence signal from anti-4E-BP1 antibody was measured. Internal normalisation control lymphoblasts were included within each assay in case of between-experiment variation.

### 4.8. Quantification and Statistical Analysis of Biochemical Assays

Data was analysed using Microsoft Excel with the WinStat add-in (Fitch, R.K., http://www.winstat.com) and R [44] using the packages R Commander [45], REzy [46], Rattle [47], pROC [36] and stats. The linear discriminant analysis (WinStat) used as prior probabilities for the proportions of the ME/CFS and control samples in the total cohort. In the logistic regression (REzy, R Commander), the propensity score was calculated from the fitted logit function. The propensity score represents a probability that the sample in question is from an ME/CFS patient. For both methods, we used either a single independent variable or a set of five key respirometric parameters that are significantly altered in ME/CFS lymphoblasts. For lymphoblast TORC1 activity, the independent variable was the logarithm of the normalised phosphorylation level of 4E-BP1, a specific cellular substrate of TORC1. For lymphocyte death rate it was the percentage of dead lymphocytes after 48 h in culture medium. For respirometry, the five key parameters used were the fraction of the basal O_2_ consumption rate (OCR) attributable to ATP synthesis by Complex V and the use of the proton gradient in other mitochondrial membrane transport processes (the proton leak), maximum CCCP-uncoupled OCR, the maximum uncoupled Complex I activity and the nonmitochondrial OCR. The outcomes of both the linear discriminant analysis and the logistic regression were expressed as a “confusion matrix”, showing a cross tabulation of the actual source of the sample (ME/CFS or control) and the classification produced by the method in question. From this, the error rates (false positives and false negatives) were calculated. 

Linear discriminant analysis allocates an individual sample to the group whose average it is closest to, on the test criteria being used, while logistic regression makes the assignment on the basis of which group the sample has the higher likelihood of belonging to (propensity score > 0.5). Although arbitrary, these criteria for determining test thresholds make intuitive sense. However, in neither case are they designed to minimise the overall error rate, so that they can yield large differences between the false positive and false negative rates. We therefore also performed Receiver Operating Characteristic (ROC) analysis on the outcomes of the logistic regression, during which the sensitivity (fraction of positives that are correct) is plotted against specificity (fraction of negatives that are correct). Since ROC analysis depends only on the rank order of observations in the data set, when there is only a single independent measure being tested, it produces identical results for the raw and for rescaled or transformed data from the corresponding linear discriminant or logistic regression analysis. Only the scale changes on which the threshold is measured. For consistency, and to facilitate comparisons between different assays, we have conducted ROC analysis on the propensity scores from logistic regression throughout this paper. The AUC (area under the ROC curve) with 95% confidence limits was calculated as an indicator of the usefulness of the biomarker in question in distinguishing ME/CFS from control samples. The “best” threshold value for the biomarker in question was defined in the ROC analysis as the point on the ROC curve which maximised the sum of the sensitivity and specificity (i.e., minimised the sum of the errors). ROC curve comparisons by the bootstrapping method were done as described by Robin et al. (2011) using 2000 replicates [36]. Confidence limits for the sensitivities (on the vertical axis of ROC curves) were used to plot 95% confidence limits for the ROC curve itself.

## 5. Conclusions

In previous work we showed that ex vivo lymphocytes from ME/CFS patients exhibit an elevated death rate in culture after recovery from frozen storage and that lymphoblastoid cell lines isolated from them exhibit multiple mitochondrial and cellular stress signalling abnormalities [32]. The objective in this preliminary investigation was to determine if these abnormalities could be used as blood-based biomarkers of disease. Our results here demonstrate that these abnormalities are, indeed, promising candidate biomarkers, each of them able to distinguish ME/CFS patient and control samples with better than 80% reliability. With each test providing very high sensitivity (correct detection of positive samples) but lower specificity (correct identification of negative samples) vís-a-vís controls, our results suggest these tests would most usefully be deployed as components of a staged protocol. The first stage involves a cheap and rapid screening test using the frozen lymphocyte death rate. The results from this could be used by clinicians and patients to decide whether to complete the 2^nd^ stage in which mitochondrial respiratory function and TORC1 signaling activity are measured in lymphoblastoid cell lines derived from the frozen lymphocytes. The combined tests may provide a highly reliable cell-based blood testing protocol to aid in ME/CFS diagnosis.

## Figures and Tables

**Table 1:** “Confusion matrix” analysis of lymphocyte death rate after 48 h in culture medium.

Method	Clinical Group	Actual Count	Test Class			% Error	Statistics	
			ME/CFS		Control			
Linear discriminant analysis	ME/CFS	57	52	5		8.8	χ^2^	25.5
	Control	33	13	20		39.4	*p*	4.5 × 10^−7^
	TOTAL	90	65	25		20.0	Fisher *p* = 2.2 × 10^−7^	
Logistic regression	ME/CFS	57	52	5		8.8	χ^2^	25.5
	Control	33	13	20		39.4	*p*	4.5 × 10^−7^
	TOTAL	90	65	25		20.0	Fisher *p* = 2.2 × 10^−7^	

**Table 2:** “Confusion matrix” analysis of lymphoblast respiratory function and mitochondrial membrane potential (ΔΨ_m_).

Method	Clinical Group	Actual Count	Test Class		% Error	Statistics	
			ME/CFS	Control			
Respiratory function + ΔΨ_m_							
Linear discriminant analysis	ME/CFS	49	46	3	6.1	χ^2^	27.9
	Control	20	6	14	30.0	*p*	1.3 × 10^−7^
	TOTAL	69	52	17	13.0	Fisher *p* = 1.2 × 10^−7^	
Logistic regression	ME/CFS	49	46	3	6.1	χ^2^	24.4
	Control	20	7	13	35.0	*p*	7.7 × 10^−7^
	TOTAL	69	54	15	14.5	Fisher *p* = 7.7 × 10^−7^	
Respiratory function only							
Linear discriminant analysis	ME/CFS	49	46	3	6.1	χ^2^	12.4
	Control	20	11	9	55.0	*p*	4.4 × 10^−4^
	TOTAL	69	57	12	20.2	Fisher *p* = 3.8 × 10^−4^	
Logistic regression	ME/CFS	49	46	3	6.1	χ^2^	15.1
	Control	20	10	10	50.0	*p*	1.0 × 10^−4^
	TOTAL	69	53	16	18.8	Fisher *p* = 9.3 × 10^−5^	

**Table 3:** “Confusion matrix” analysis of lymphoblast TORC1 activity.

Method	Clinical Group	Actual Count	Test Class		% Error	Statistics	
			ME/CFS	Control			
Linear discriminant analysis	ME/CFS	44	40	4	9.1	χ^2^	16.6
	Control	22	9	13	40.9	*p*	4.5 × 10^−5^
	TOTAL	66	49	17	19.7	Fisher *p* = 2.9 × 10^−5^	
Logistic regression	ME/CFS	44	40	4	9.1	χ^2^	16.6
	Control	22	9	13	40.9	*p*	4.5 × 10^−5^
	TOTAL	66	49	17	19.7	Fisher *p* = 2.9 × 10^−5^	

**Table 4:** “Confusion matrix” analysis of combined tests.

Method	Clinical Group	Actual Count	Test Class		% Error	Statistics	
			ME/CFS	Control			
Linear discriminant analysis	ME/CFS	29	28	1	3.4	χ^2^	31.7
	Control	14	1	13	7.1	*p*	1.8 × 10^−8^
	TOTAL	43	29	14	4.7	Fisher *p* = 5.2 × 10^−9^	
Logistic regression	ME/CFS	29	28	1	3.4	χ^2^	31.7
	Control	14	1	13	7.1	*p*	1.8 × 10^−8^
	TOTAL	43	29	14	4.7	Fisher *p* = 5.2 × 10^−9^	

**Table 5:** “Confusion matrix” analysis of combined tests for participants whose lymphocytes had been tested for the rate of cell death in culture.

Method.	Clinical Group	Actual Count	Test Class		% Error	Statistics	
			ME/CFS	Control			
“ME/CFS suspected”							
Combined test	ME/CFS	27	27	0	0	χ^2^	3.00
	Control	2	1	1	50	*p*	0.083
	TOTAL	29	28	1	3.4	Fisher *p* = 0.069	
“ME/CFS not suspected”							
Combined test	ME/CFS	2	1*	1	50	χ^2^	1.12
	Control	12	0	12	0	*p*	0.29
	TOTAL	14	1	13	7.1	Fisher *p* = 0.14	

*Indicates the single individual who would have been “missed” in the triage screening of a staged protocol but subsequently correctly diagnosed in the combined tests.

**Table 6:** “Confusion matrix” analysis of respirometry and TORC1 activity tests for participants whose lymphocytes had not been tested for the rate of cell death in culture.

Method	Clinical Group	Actual Count	Test Class		% Error	Statistics	
			ME/CFS	Control			
Respirometry test	ME/CFS	16	15	1	6.3	χ^2^	5.96
	Control	6	2	4	33.3	*p*	0.015
	TOTAL	22	18	4	13.6	Fisher *p* = 9.3 × 10^−3^	
TORC1 activity test	ME/CFS	15	14	1	6.7	χ^2^	0.352
	Control	8	6	2	75.0	*p*	0.553
	TOTAL	23	20	3	30.4	Fisher *p* = 0.269	

## Appendix A

**Table A1:** Confusion matrix for linear discriminant analysis of PBMC death rate after 24, 48 and 72 h in culture medium.

Method	Clinical Group	Actual Count	Test Class		% Error	Statistics	
			ME/CFS	Control			
Linear discriminant analysis	ME/CFS	57	45	12	21.0	χ^2^	26.2
	Control	33	7	26	21.2	*p*	3.0 × 10^−7^
	TOTAL	90	52	38	21.1	Fisher *p* = 1.25 × 10^−7^	
Logistic regression	ME/CFS	57	47	10	17.5	χ^2^	27.4
	Control	33	8	25	24.2	*p*	1.6 × 10^−7^
	TOTAL	90	65	25	20.0	Fisher *p* = 7.2 × 10^−8^	

**Table A2:** Confusion matrix for logistic regression of PBMC and lymphoblast ME/CFS biomarkers using a randomly selected training and test subcohorts.

Subcohort	Clinical Group	Actual Count	Test Class		% Error	Statistics	
			ME/CFS	Control			
PBMC cell death after 48 h in culture							
Training	ME/CFS	46	41	5	10.9	χ^2^	17.0
	Control	25	10	15	40.0	*p*	3.8 × 10^−5^
	TOTAL	71	51	20	21.2	Fisher *p* = 2.1 × 10^−5^	
Test	ME/CFS	11	11	0	0	χ^2^	6.39
	Control	8	3	5	37.5	*p*	0.012
	TOTAL	19	14	5	15.8	Fisher *p* = 4.8 × 10^−3^	
Lymphoblast Seahorse respirometry							
Training	ME/CFS	37	34	3	8.1	χ^2^	16.6
	Control	17	6	11	35.3	*p*	4.6 × 10^−5^
	TOTAL	54	40	14	16.6	Fisher *p* = 3.1 × 10^−5^	
Test	ME/CFS	12	10	2	16.7	χ^2^	4.22
	Control	3	0	3	0.0	*p*	0.04
	TOTAL	15	10	5	13.3	Fisher *p* = 0.022	
Lymphoblast TORC1 activity							
Training	ME/CFS	34	30	4	11.8	χ^2^	10.65
	Control	19	8	11	42.1	*P*	1.1 × 10^−3^
	TOTAL	53	38	15	22.6	Fisher *p* = 9.1 × 10^−4^	
Test	ME/CFS	10	9	1	10.0	χ^2^	1.59
	Control	3	1	2	33.3	p	0.21
	TOTAL	13	10	3	15.3	Fisher *p* = 0.11	
Combined tests							
Training	ME/CFS	22	21	1	4.5	χ^2^	20.9
	Control	11	1	10	9.1	*p*	4.9 × 10^−6^
	TOTAL	33	22	11	6.1	Fisher *p* = 1.3 × 10^−6^	
Test	ME/CFS	7	6	1	14.3	χ^2^	3.35
	Control	3	0	3	0.0	*p*	0.067
	TOTAL	10	6	4	10.0	Fisher *p* = 0.033	
